# Influence of Recycling and UV Exposure on the Properties of 3D Printing Polymer Materials

**DOI:** 10.3390/polym16233292

**Published:** 2024-11-26

**Authors:** Jolanta Janutėnienė, Marius Vasylius, Artūras Tadžijevas, Valentinas Kartašovas, Deivydas Šapalas, Simona Grigaliūnienė

**Affiliations:** 1 Department of Engineering, Klaipeda University, Bijunu St. 17, LT-91224 Klaipeda, Lithuania; simona.grigaliuniene@ku.lt; 2 Marine Research Institute, Universiteto Av. 17, LT-92295 Klaipeda, Lithuania; marius.vasylius@ku.lt (M.V.); arturas.tadzijevas@ku.lt (A.T.); deivydas.sapalas@ku.lt (D.Š.)

**Keywords:** 3D printing, ABS, PLA, PET-G, tensile test, impact test, UV, tensile strength, relative elongation, degradation of mechanical properties

## Abstract

The use of polymer materials in various fields has increased significantly due to their ease of thermoforming and relatively low production costs. The production volume of these materials is extremely high, and according to forecasts from global statistical centers, it is expected to continue rising in the future. However, the extensive use and easy availability of polymeric materials have caused significant ecological problems. The world faces large amounts of polymer waste and environmental pollution. Plastic recycling remains challenging due to issues related to sorting polymer waste and separating it according to polymer types. Recycling certain plastics requires only a quarter of the energy needed to produce new plastic. To address this, circular economy principles should be applied to 3D printing products made from polymeric materials. A particularly wide application of these technologies is found when polymeric materials are used due to their low cost, low melting temperatures, and other advantageous properties. This paper investigates the impact of plastic recycling on the quality of 3D-printed products. During the research, samples were 3D printed and tested using both virgin and recycled PLA, ABS, and PET-G materials. The samples underwent static and dynamic tests to determine their mechanical properties, such as tensile strength, elongation, and impact resistance. The research results showed that the properties of recycled polymer materials deteriorate, with relative elongation of recycled and 3D-printed materials decreased by 16–45%. Despite this, recycled polymer materials can still be used, but it is necessary to account for the reduction in plasticity when creating products that will be exposed to dynamic loads. The impact strength is reduced by 6% for PLA, 54% for ABS, and 58% for PET-G. Additionally, the research included tests on samples printed with 3D printing technology that were exposed to UV irradiation. The results indicated similar dependences, as UV exposure also affects the reduction of material plasticity. After 66 Wh/m^2^ of UV radiation, the tensile strength of PET-G and PLA decreased by 17%, while ABS showed a reduction of about 5%.

## 1. Introduction

Humanity is currently facing major environmental, social, and economic problems around the world. To address these global issues at the international cross-border level and create a more sustainable and better future for all, the United Nations adopted 17 Sustainable Development Goals (SDGs) in 2015 [1]. Three-dimensional printing technology offers the opportunity to customize production methods while reducing material waste. The sustainability of 3D printing is crucial as it becomes increasingly significant in the manufacturing market. The use and development of recyclable, biodegradable, and sustainable materials for extrusion 3D printing can reduce environmental impact and demonstrate the importance of this technology as a sustainable manufacturing method [2]. The use of biodegradable materials in 3D printing has gained attention due to their potential to address environmental issues in the manufacturing industry. Biodegradable materials, such as bioplastics, are increasingly being used in 3D printing as an eco-friendly alternative to traditional materials [3]. Some of the material needs of additive manufacturing include sustainable printing inks, sources of resins and filaments, and ways to process, recycle, and chemically transform polymers. In addition, combining bio-sourced and biodegradable polymers with additive manufacturing could produce objects that, after performing their function, can be recycled back into raw materials or degraded into non-toxic products [4].

Research on current trends in the use of recycled materials in the AM process in-volves converting waste materials into stereolithography (SLA) and digital light projection (DLP) printer resins, fused deposition modeling (FDM) filaments, among others [5].

Three-dimensional printing technology has created a different approach to manufacturing, transforming the manufacturing process by reducing raw material waste [6,7]. Three-dimensional printing involves various technologies that differ significantly from each other and have unique characteristics from the material used for the casting method. Each of these technologies is applicable to different materials and industries. According to ASTM standards, there are seven groups of 3D printing: binder spraying, direct energy deposition, material extrusion, material jetting, powder bed fusion, sheet lamination, and vat photopolymerization [8]. Three-dimensional printing must be use with materials that meet high-quality requirements. Each printing technology can be used to create functional products with a wide choice of materials, the most common being metals, polymers, ceramics, and composite materials [9].

The most common plastics used for 3D printing are PLA (polylactide) and ABS (acrylonitrile, styrene, and polybutadiene copolymer). Special filaments for printing are made from these materials. ABS has a higher glass transition temperature compared to many other common plastics and can withstand much higher temperatures before it begins to deform [10]. Additionally, this plastic is characterized by heat and chemical resistance. ABS mixed with nylon was one of the first composites used in the history of FDM [11].

One of the most promising materials for 3D printing is PLA, derived from renewable resources, such as corn starch and sugar cane. PLA is described as a bio-material suitable for many healthcare applications, such as tissue engineering and regenerative medicine. PLA is the most popular 3D printing material, with significant applicability in various fields, including medicine, food packaging, prototyping, and more [12]. PLA has become a preferred material because it is eco-friendly (biodegradable), biocompatible (non-toxic), and processable (with better thermal properties). The properties of PLA give the polymer many advantages over other conventional polymers such as ABS (acrylonitrile butadiene styrene).

Three-dimensional-printed plastic products must meet user requirements and be of high quality. Along with the advancement of 3D printing technology, there has also been an increase in research into how to ensure the quality of printed products. This is especially important when producing parts for devices where operational conditions are critical.

Three-dimensional printing technologies using polymer filaments were initially limited to the production of prototypes. Later, they were applied to the production of finished functional products with excellent mechanical properties. However, this technology has drawbacks that are important for the assembly and service life of functional parts. Geometric properties often fall short of the optimal level obtained by traditional manufacturing processes [13]. In [14], it was established that micro-level defects in 3D-printed parts have an influence on mechanical properties. Changes in the properties of polymeric materials are also observed when using laser technologies for printing [15,16].

The research conducted includes studying the influence of printing conditions, colors, printing steps, and printing direction on the mechanical characteristics of printed materials. Pandzic et al. [17] found that the color of the PLA specimen affects the modulus of elasticity, yield strength, ultimate stress, hardness, and deformation.

The influence of the layer thickness on the mechanical properties of the printed ABS samples was investigated in [18]. The filling level and shape of the specimens have a significant influence on tensile strength. Specimens with 25% infill (higher porosity) withstand a lower maximum load than samples with a higher percentage of filling (50% and 100%) and lower porosity. Infill octagon-type specimens have the best tensile strength compared to straight-shaped specimens [19]. In response to the principles of the circular economy and the development of 3D printing technologies, recycled plastic materials for 3D printing are already being produced, such as rABS (recycled ABS), rPP (polypropylene), rPET (polyethylene terephthalate), and rPLA (see, for example, [20,21]).

UV aging significantly affects the mechanical properties of plastics. It has been observed that the stress at fracture and deformation at fracture of original materials are much higher than those of aged materials. For example, these properties decrease dramatically after 72 h of UV exposure and then stabilize with prolonged exposure. Specifically, after 72 h, the stress decreased by about 39%, and the deformation by about 61%; while after 216 h, the stress and deformation were reduced by 47% and 64%, respectively [22]. Vasylius et al. [23] found that UV aging has a significant effect on the mechanical properties of PET films, including a loss of ductility even after short exposure to solar radiation. UV exposure caused a deterioration in the mechanical properties of A-PET material, with a greater effect observed in aged material. Rocket et al. [24] provided a general review of the effects of UV radiation on various materials and found that organic additives can be added to polymers at much lower concentrations than inorganic fillers to achieve the desired UV stabilization.

The aim of the literature review was primarily to analyze research that investigates the properties of samples made from various polymer materials produced by 3D printing. In [25], the mechanical behavior of printed ABS with different infill densities was investigated. Researchers confirmed the hypothesis that a higher infill density in ABS leads to fewer voids in the material, resulting in increased tensile strength. Additionally, the effect of test speed on mechanical properties was investigated, revealing that ABS exhibits either ductile or brittle behavior depending on the load speed. A comparative analysis of the tensile mechanical properties of ABS obtained by FDM printing and casting was carried out in [26]. Specimens were 3D printed in three different orientations (0°, 45°, 90°), and the influence of temperature on tensile strength was analyzed. It was deduced that the injection molded ABS has a 24% higher value of tensile strength than the FDM printed ABS samples.

In [27], the influence of temperature on the properties of 3D-printed specimens made from PLA with 100% infill was investigated. Specimens were exposed to temperatures of 70–80 °C for 10–13 days, and their mechanical properties were investigated. It was deduced that heat treatment at temperatures close to the glass transition temperature of the PLA polymer improved the adhesion strength between the printed layers. In [28], tests of 3D-printed samples from PLA and recycled PLA were performed, and their properties were determined, including tensile strength and three-point bending strength. The tests varied the thickness of the printed layer (0.15–0.25 mm) and the infill rate (30–70%). The results confirmed that recycling PLA affects the mechanical properties. Specifically, PLA exhibited stronger tensile strength than recycled PLA at all layer thick-nesses and infill rates. Research on 3D-printed nylon material reinforced with fiber was carried out in [29]. Experimental investigations of 3D-printed PLA samples obtained using FDM printing were carried out in [30]. The specimens were printed at different angles (0°, 45°, 90°) with varying layer thicknesses. The influence of these parameters on the main mechanical properties was investigated. It was observed that increasing the thickness of layers increases Young’s modulus and tensile strength of material.

Research on recycled PLA materials was conducted in [31]. It was found that the mechanical properties of 3D-printed specimens made from recycled PLA fiber were like those of the original specimens. Specifically, the flexural strength of specimens recycled once and twice (106.8 ± 9.0 MPa and 108.5 ± 9.9 MPa, respectively) was comparable to the strength of the original specimens (119.1 ± 6.6 MPa). However, the third recycling process adversely affected the strength values, resulting in a significant reduction (75.0 ± 16.2 MPa). Rigon et al. [32], presented a review of properties of recycled ABS and PP materials produced by injection molding. It was found that the static properties of recycled ABS are like those of the original material. However, the tensile strength of recycled PP material is reduced by approximately 40%.

The change in the mechanical properties of the polymer material PVC and the polymer composite with glass fiber due to the effect of ultraviolet (UV) rays was investigated in [33].

Recycled polymer materials are increasingly used in the production of 3D-printed products, but the quality of these products is only fragmentarily studied. It is not well understood how much the material’s properties change after use, UV exposure, recycling, and 3D printing. This is particularly relevant in cases where critical products or parts are produced that are subject to external static and dynamic loads. Gomes et al. [34] analyzed papers and made general observations on settings that affect the properties of printed parts with recycled materials, including part orientation, raster angle, nozzle diameter, layer height, infill ratio, wall structure, and printing speed.

Recycled polymer materials for 3D printing are investigated in this work. Three commonly used materials for printing industrial and household products were selected for this study: ABS, PLA, and PET-G (polyethylene terephthalate glycol). The properties of both new and recycled materials were compared.

Our research observed that the properties of polymeric materials deteriorate and become more brittle after prolonged storage under normal room conditions. To further investigate this, samples of printed materials were exposed to UV light. The results allowed us to determine the minimum duration of UV exposure required for significant changes in the materials’ properties.

## 2. Materials and Methods

The purpose of this study is to determine and compare the mechanical properties of new and 100% recycled PLA, ABS, and PET-G materials for 3D printing, as well as to evaluate the degradation rate of these properties under UV exposure. Thermoplastics, such as PLA, are easily injection molded and can then be recycled [12]. This study used certificated polymer filaments for 3D printing obtained from the same commercial manufacturer who supplies both new and recycled materials.

In today’s manufacturing and industrial sectors, it is important to focus on long-term technological sustainability and plan the lifecycle to predict failure. Therefore, the properties of recycled R_PLA, R_ABS, and R_PET-G are investigated during this research. Analyzing the conducted scientific studies, positive trends can be observed in terms of the properties of recycled plastics, as well as various recommendations for improving these properties. Additionally, there are increasing adaptability properties. Recycling PLA waste is considered an acceptable solution that reduces the amount of PLA waste in landfills and the environment [35]. It has also been analyzed that, by modifying the composition of R-PET and polycarbonate (PC) composites, the mechanical properties of the material can be improved [36].

Materials. During this research, samples were printed from different polymer mate-rials: PLA, ABS, PET-G, R_PLA, R_ABS, and R_PET-G filaments. Both new and recycled materials were purchased from specialty stores that sell manufacturer-certified 3D printing filaments. Manufacturers of 3D printing materials provide information about the waste used to produce the printing filament. Recycled materials can be made from residual extrusion waste streams, recycled food packaging waste, or other waste sources. This study used 100 percent recycled material, which does not contain any additional additives. These materials are most often used in households and industry. The use of recycled polymer materials helps reduce the carbon footprint of commercial products [37]. Different materials have distinct properties, so it is necessary to consider certain advantages when choosing the required type of material. PLA is one of the most widely used materials in 3D printing and can be used to create various parts and prototypes for industries, such as medicine, food, cosmetics, and textiles, among others. Although PLA is classified as a more environmentally friendly material, it is short-lived when exposed to environmental conditions, such as moisture and UV light. ABS is a non-biodegradable thermoplastic, impact-resistant, lightweight, and can be painted and machined. It is suitable for use from −40 °C to 80 °C but is sensitive to direct sunlight. PET plastic has a very low and stable coefficient of friction, so it wears longer compared to other types. Additionally, it can operate at higher temperatures and is more resistant to various acids.

Three-dimensional Printing. The test printing technology used is FDM (Fused Deposition Modeling). The printing device is an Original Prusa MK4 with a standard print head. The nozzle hole diameter is 0.4 mm, and the printing width is 112.5% of the nozzle diameter, i.e., 0.45 mm. The infill density is 100 percent, and the layer thickness is 0.2 mm. Infill pattern is rectilinear, infill patterns change by layers 0°–90°, infill/perimeter overlap is 25%. Perimeters—2, top and bottom layers—2. Flowrate perimeters, outer layer is 95%. Flowrate infill 95%. Environmental temperature 23°. Printing speed initial layer 70 mm/min. Printing speed perimeters 45 mm/min. Printing speed infill 200 mm/min. Printing speed top layer 30 mm/min. Filament retracking 0.8 mm. The printer’s printing parameters for different materials are given in Table 1.

Investigation of Mechanical Properties. The static mechanical properties investigated include the modulus of elasticity, tensile strength, breaking point, and elongation at the breaking point.

**Figure 1:**
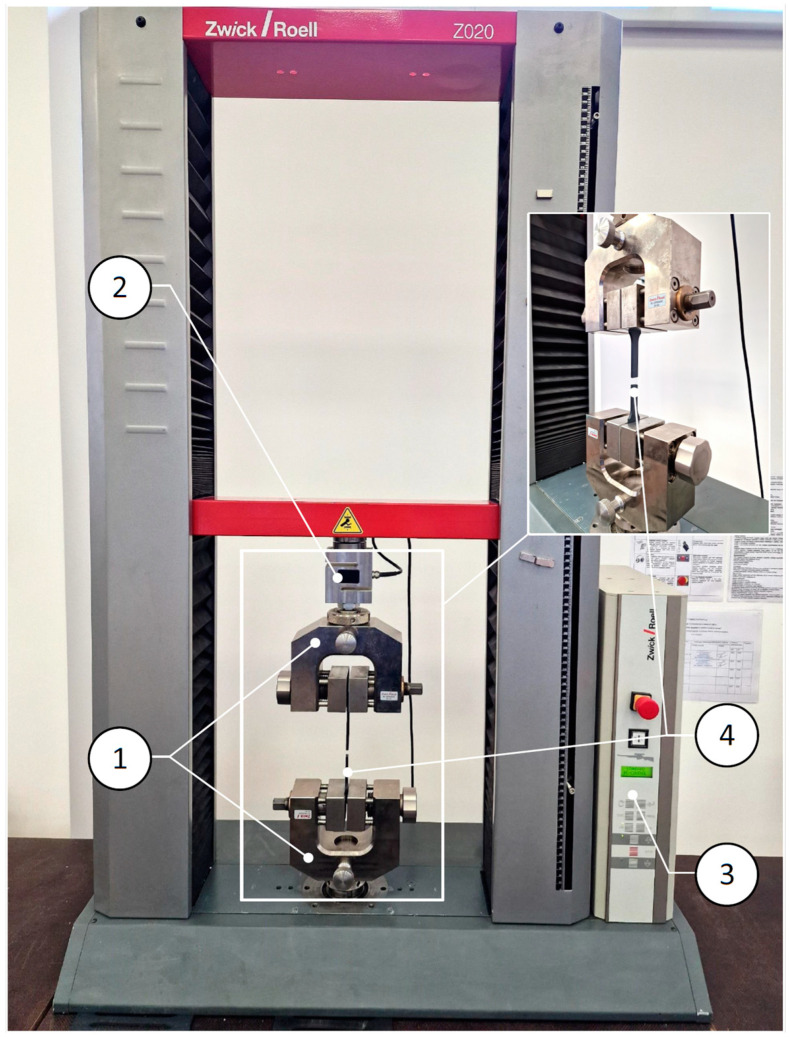
Universal testing machine Zwick/Roell Z020: 1—specimen-holding clamps “GRIPS”; 2—20 kN force cell; 3—machine controller; 4—specimen.

A Zwick/Roell Z020 testing machine with wedge grips was used for the tests (Figure 1). Research data were analyzed using testXpert III software.

**Figure 2:**
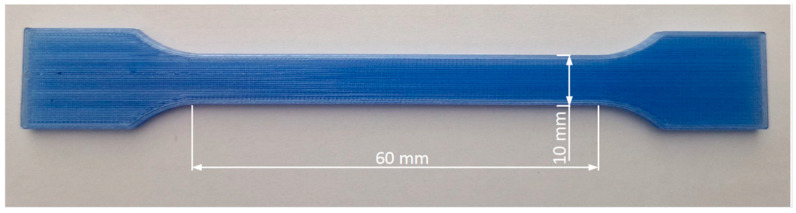
Specimen for a tensile test.

Tensile samples (Figure 2) were printed according to ISO 527-2 [38], sample type B. The length of the sample is 150 mm, and the working part of the sample is 60 mm. The width of the sample is 10 mm, and the thickness is 4 mm. The tensile test speed was set to 10 mm/min.

**Figure 3:**
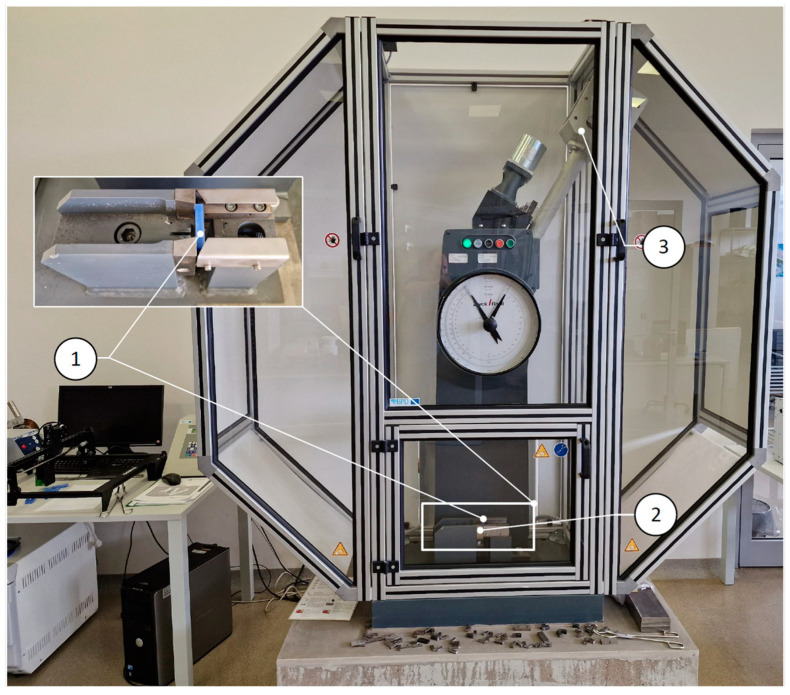
Dynamic testing machine Zwick/Roell RKP450. 1—specimen, 2—grip., 3—pendulum.

Investigation of Dynamic Mechanical Properties. The Charpy impact test was used as the primary test method. A Zwick/Roell RKP450 pendulum testing machine (Figure 3) was utilized for dynamic testing. Additionally, to provide more detailed testing of the dynamic characteristics of the materials, a Zwick/Roell Amsler HIT200F drop-weight testing machine (with 150 J energy and an initial impact velocity of 5.23 m/s) was used. This allowed for the determination of the elastic and plastic zones of the energy required to break the specimens.

**Figure 4:**
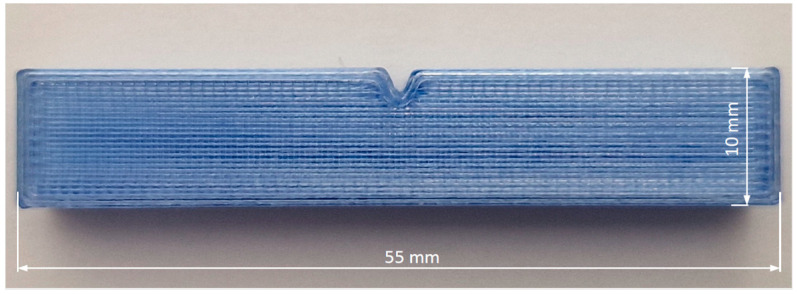
Specimen for impact test.

Impact sample is shown in Figure 4. The length of the samples is 55 mm, the width is 10 mm, and the thickness is 10 mm. The notch is V-shaped, 2 mm deep with a rounding radius of 0.25 mm.

**Figure 5:**
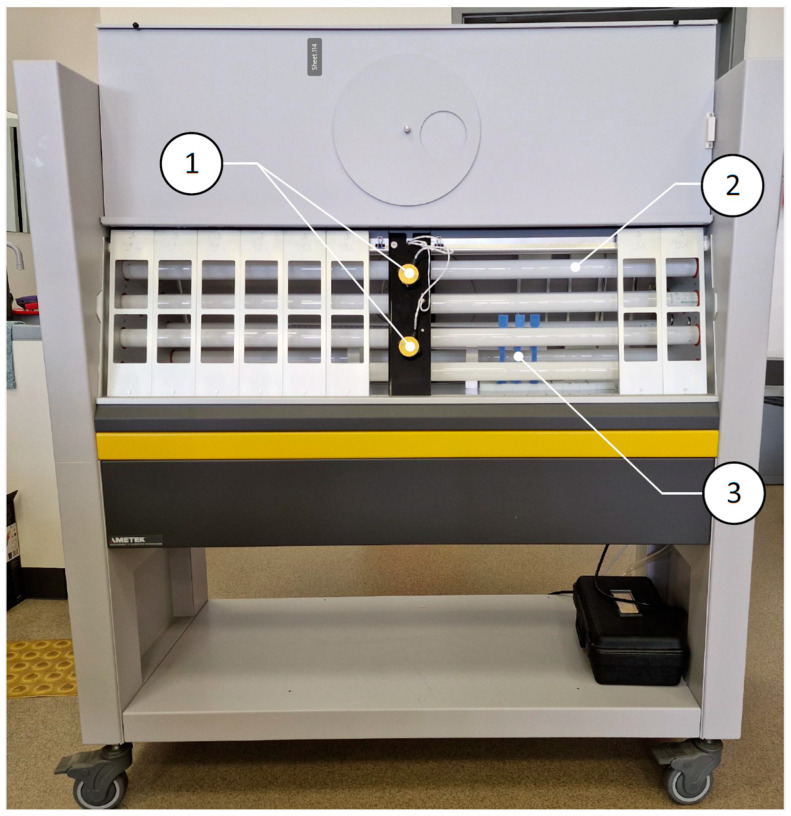
UV-aging chamber Atlas UV Test: 1—UV calibration and intensity sensors; 2—UV lamp; 3—specimen.

UV Exposure Investigation. A UV-aging chamber, Atlas (Figure 5), equipped with temperature and humidity control functions, was used for UV exposure.

## 3. Results and Discussion

### 3.1. Mechanical Properties of 3D Printed Recycled Polymer Materials

Tensile tests were performed to derive the influence of recycling on the polymer materials PLA, ABS, and PET-G. Ten specimens for each material test were printed and tested. The mean, standard deviation, and coefficient of variation were calculated to show the dispersion of the results.

**Figure 6:**
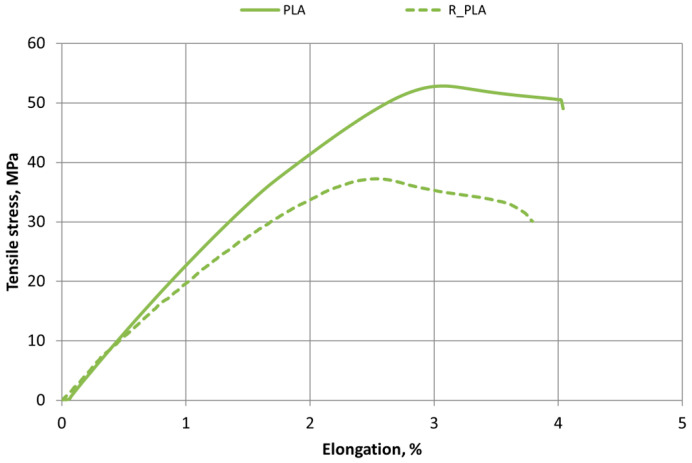
Stress–strain diagrams of printed PLA and R_PLA materials.

**Figure 7:**
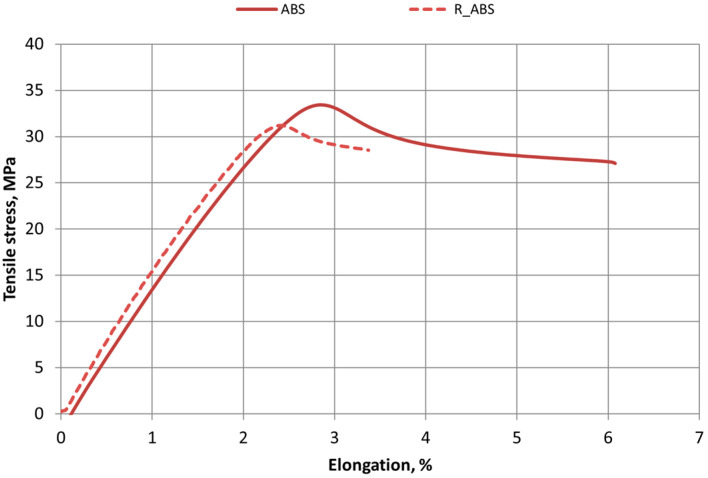
Stress–strain diagrams of printed ABS and R_ABS materials.

**Figure 8:**
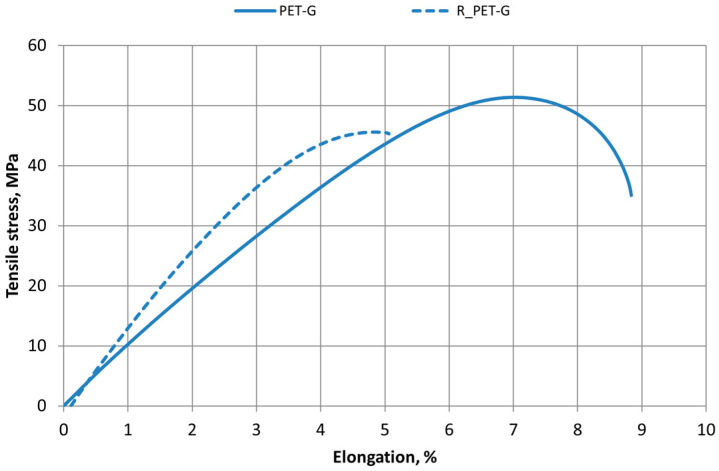
Stress–strain diagrams of printed PET-G and R_PET-G materials.

Figure 6, Figure 7 and Figure 8 show the tensile test stress–strain diagrams of 3D-printed specimens made from PLA, ABS, and PET-G new and recycled materials. In this study, it was observed that the elongation of the new materials is higher than that of the recycled materials, indicating that the recycled material specimens are less ductile. It was also observed that the tensile strength of recycled materials is also lower. The tensile strength and elongation characteristics of all materials used in this study are presented in Table 2 and Table 3.

Decreasing values of tensile strength and relative elongation were observed in all materials—PLA, ABS, and PET-G.

Analyzing the properties of 3D-printed ABS materials in the scientific literature under similar 3D printing conditions (room temperature, 100% infill), the tensile strength values of the material range from 29 to 42 MPa [25,26,31]. The range of PLA tensile strength values under the same printing conditions varies from 39 to 51 MPa [27,39].

Tensile test results showed that 3D printing changes the mechanical properties of materials and can cause variations of about 10 percent. It has been noted that this variation can be affected by the length of time between the production of the print filament and its use for 3D printing. So, the same coil material should be used for reliable results.

The recycling process affects changes in the mechanical properties of polymeric materials.

**Figure 9:**
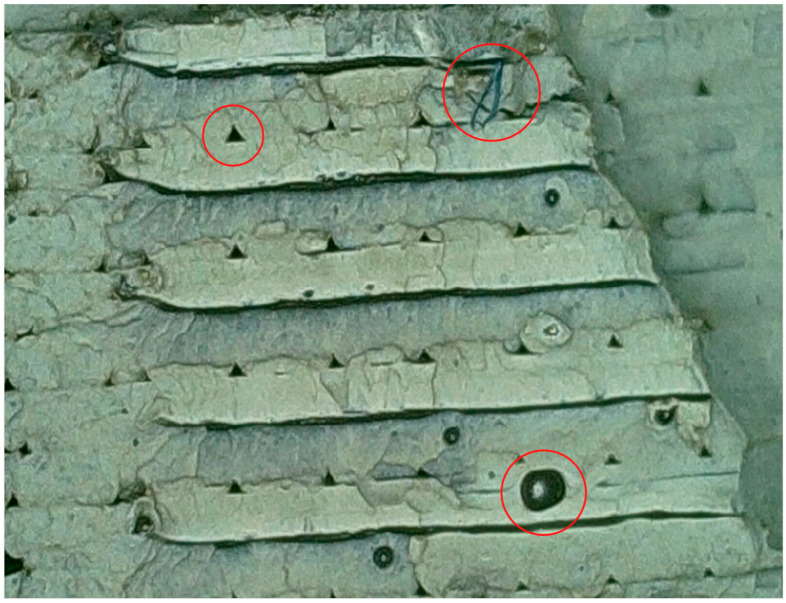
View of fracture surface of specimen of recycled material R_ABS.

In Figure 9, the view of fracture of R_ABS specimen is presented after impact testing. We can see in red circles printing voids, defects, and the inclusion of extraneous materials.

Tensile test results for new materials and recycled polymer materials are presented in Table 2, which compares the tensile strength values of all tested materials: PLA, R_PLA, ABS, R_ABS, PET-G, and R_PET-G. The values of the relative elongation of all material samples at Fmax are given in Table 3.

**Figure 10:**
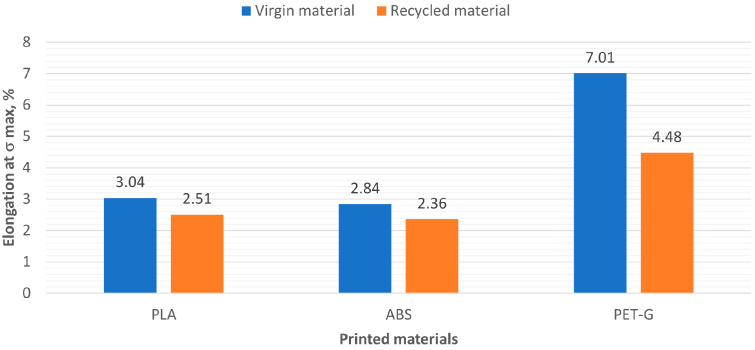
Values of relative elongation at $\sigma^{max}$ of virgin and recycled polymer materials.

Figure 10 presents a comparison of the relative elongations at $\sigma^{max}$ of virgin and recycled materials.

Three-dimensional printing changes the properties of polymer materials, the tensile strength and relative elongation values of the materials may decrease by about 10–15 percent from their initial properties before printing. Printing parameters could affect the properties of printed parts [34]. Increasing nozzle temperature, wall structure, and infill ratio have a positive effect on relative elongation and tensile strength. The author of [14] also identified that there are several defects in 3D-printed parts at micro level that have a large impact on mechanical properties.

In conducting research on the properties of 3D-printed materials, it has been observed that the properties of polymeric materials begin to deteriorate when stored for a long time in natural room conditions with varying humidity, light, etc. Polymeric materials become more brittle, and their tensile strength and relative elongation values change.

Mechanical properties of polymer materials change plasticity after recycling. The research results confirm findings by other scientists that recycling affects mechanical properties. As stated in [5], “recycled PLA specimens showed lower mechanical properties compared to virgin material”.

This tendency can be observed by analyzing the energy of the elastic and plastic zones. In Table 4, we can see that the energy of the plastic regions in recycled materials R_PLA, R_ABS, and R_PET-G is less than in virgin materials.

**Figure 11:**
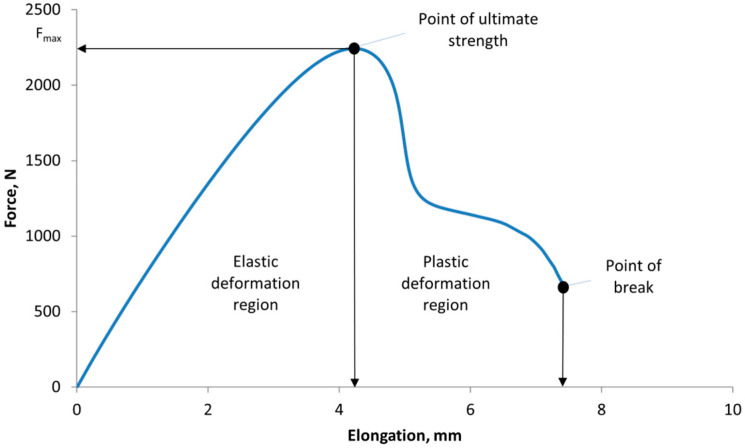
Force–deformation curve demonstrating elastic and plastic regions, and ultimate strength.

The degradation of the mechanical properties of the 3D-printed material is also well demonstrated by the calculated values of the energies required to break the samples. The tensile machine measures the elongation of the specimen and evaluates the increasing force, so the area of the tensile curve up to the maximum measured force is the area of elastic deformation, and the area from F_max_ to failure of the specimen is the area of plastic deformation Figure 11.

**Figure 12:**
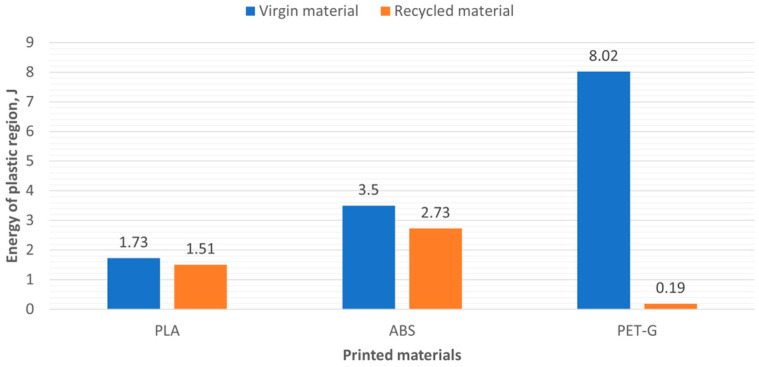
The energy in the plastic region of polymer materials.

Comparison of results of energy in the plastic region of virgin and recycled polymer materials is presented in Figure 12.

As shown in Figure 12, the energy in the plastic region is lower for recycled polymer materials compared to their virgin counterparts. Among the recycled materials, R_PET-G exhibits particularly low energy values.

### 3.2. Impact Test

**Figure 13:**
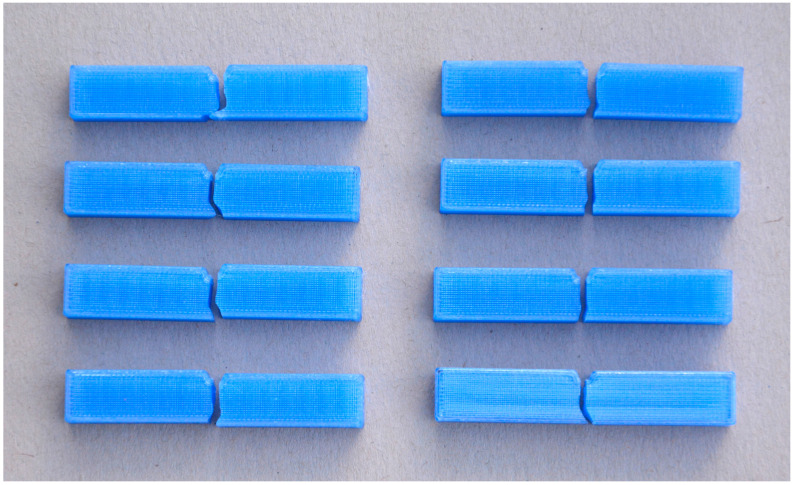
Image of fracture specimens R_PET-G after impact test.

From the investigated materials, 10 samples were printed to determine impact strength using the Charpy method. The energy required to break the specimen was measured during the test. The impact energy is calculated using Equation:

Charpy Impact Strength = E/bh 10^3^ kJ/m^2^ (1)

where E is the absorbed impact energy (Joule), b is the width of the specimen up to the notch base (mm), and h is the specimen thickness (mm). Figure 13 shows the image of the specimens after the test. As we can see, the 3D-printed specimens are characterized by brittle disintegration without plastic deformation.

**Figure 14:**
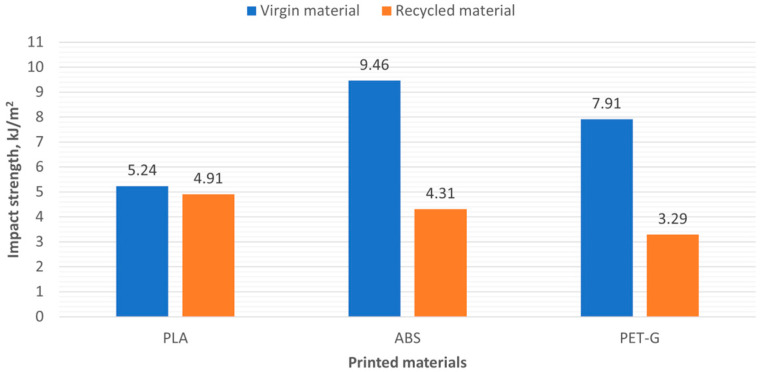
Impact strength of printed new and recycled materials.

The impact test results obtained are presented in Table 5 and Figure 14. Comparing the 3D printed samples from new and recycled materials, we observe significant differences in values. Samples made from recycled materials show lower resistance to dynamic loads. Specifically, the impact strength is reduced by 6% for PLA, 54% for ABS, and 58% for PET-G.

**Figure 15:**
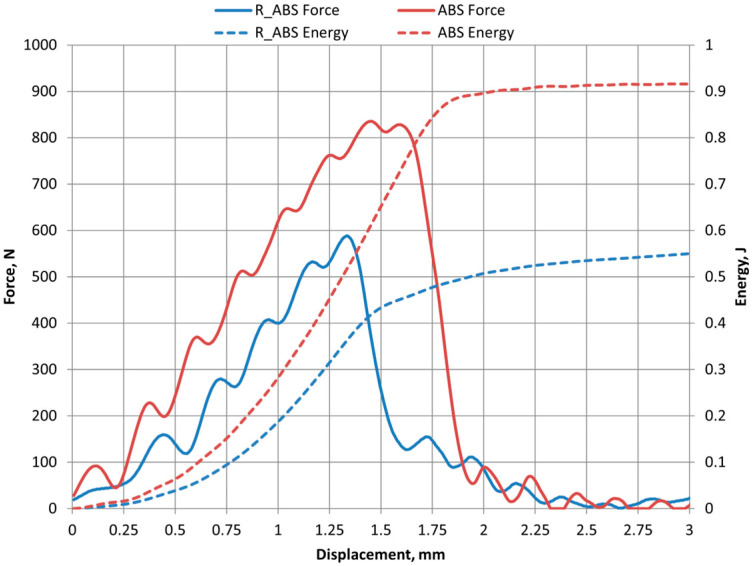
ABS and R_ABS results of Charpy impact test.

The impact test results of force and energy of ABS and R_ABS are presented in Figure 15.

As we can see, the force and energy values of the recycled material R_ABS are lower, indicating that the material is more ductile.

### 3.3. UV Effects on the Mechanical Properties of 3D Printed Polymer Materials

**Figure 16:**
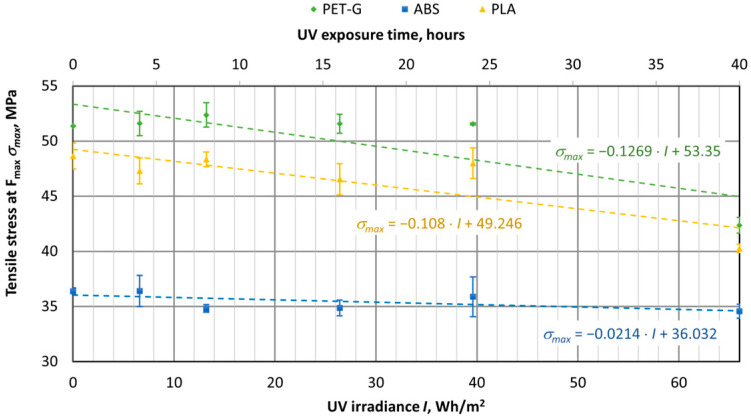
Dependences of tensile stress in 3D-printed materials on the amount of UV irradiation.

**Figure 17:**
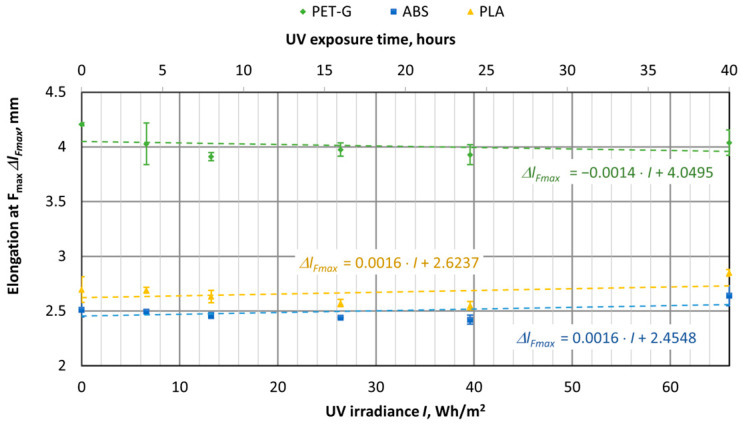
Dependences of elastic deformation of polymer 3D-printed materials on the amount of UV irradiation.

**Figure 18:**
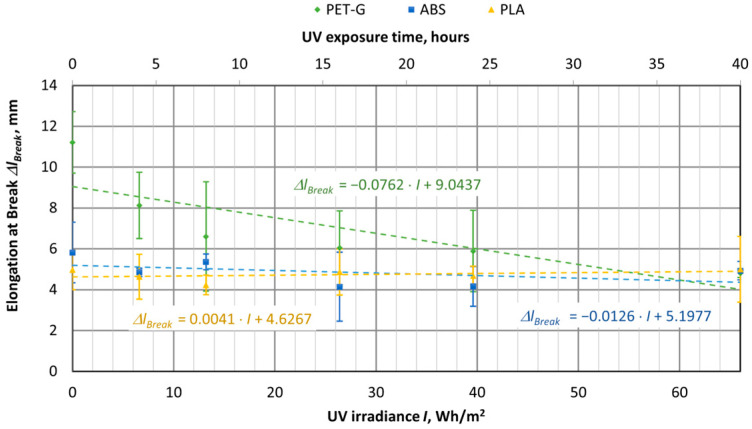
Dependences of elastic deformation of 3D-printed materials on the amount of UV irradiation.

**Figure 19:**
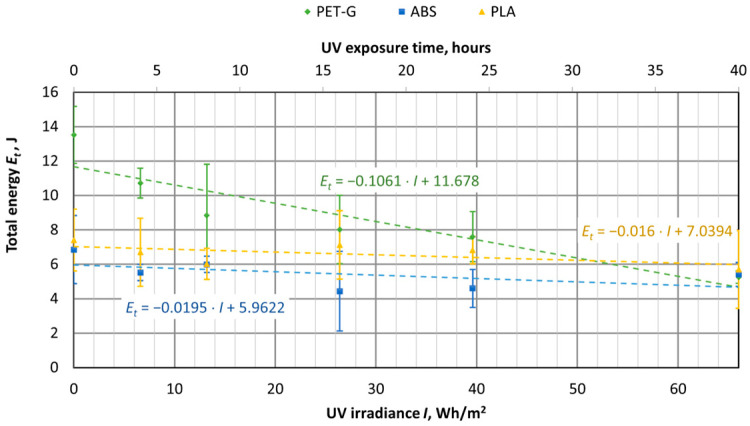
Dependences of the amount of UV irradiation on the full energy required to break the specimens with different polymer materials.

**Figure 20:**
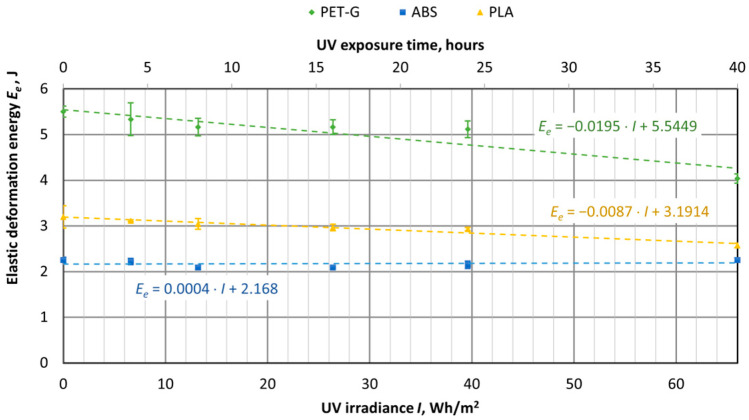
Dependences of the energy used for elastic deformation of 3D-printed materials on the amount of UV irradiation.

**Figure 21:**
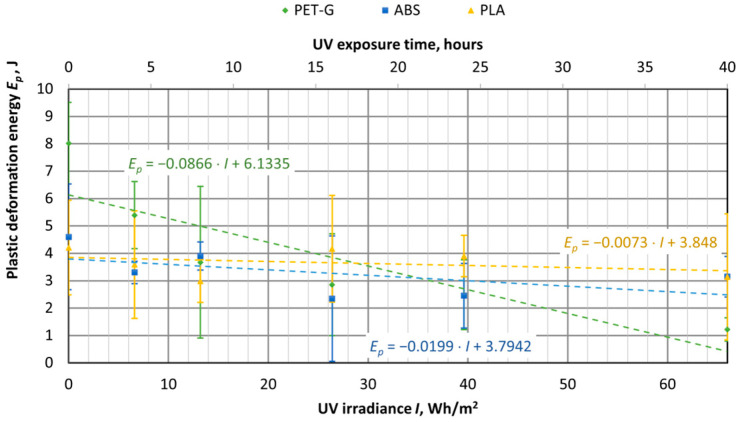
Dependences of the energy used for elastic deformation of 3D-printed materials on the amount of UV irradiation.

UV aging tests were conducted on materials used for 3D printing, using specimens of virgin PET-G, ABS, and PLA materials. The samples were exposed to UV aging in a chamber for durations of 4, 8, 16, 24, and 40 h. The tensile test results were then compared with samples that had not been exposed to UV aging (corresponding to 0 h or 0 UV radiation in the graphs). The UV aging chamber operated at an irradiance of 1.65 W/m^2^, and in Figure 16, Figure 17, Figure 18, Figure 19, Figure 20 and Figure 21, the *X*-axis represents the resulting UV irradiance in Wh/m^2^, corresponding to the exposure time in hours.

As shown in Figure 16, PET-G and PLA are affected by UV aging, while the tensile strength of ABS experiences minimal change. After 40 h of UV aging (equivalent to 66 Wh/m^2^ of UV radiation), the tensile strength of PET-G and PLA decreased by 17%, while ABS showed a reduction of about 5%

UV irradiation does not significantly affect the elastic deformation of the samples (Figure 17). However, the change in plastic deformation is quite pronounced in the PET-G material, as clearly visible from the elongation of the sample upon breaking (Figure 18). Both new and UV-aged PET-G specimens were quite ductile, with an elongation of 11.21 mm. After 40 h of aging (66 Wh/m^2^), the specimen elongated by only 4.82 mm, indicating a 57% decrease in elongation.

As shown in Figure 19, UV irradiation had the greatest impact on PET-G samples, where the energy required to break the sample decreased by 61%, from 13.52 J to 5.26 J. Meanwhile, the total energy of ABS and PLA samples also decreased by 21% and 23%, respectively. As we can see from Figure 20 and Figure 21, the reduction in energy is primarily due to the loss of plasticity in the material. This is particularly evident in the PET-G samples (Figure 18), where the energy used for plastic deformation dropped from 8.02 J to 1.22 J. The energy used for elastic deformation also decreased slightly, from 5.5 J to 4.04 J.

UV aging did not affect the amount of energy used for elastic deformation in ABS material samples. However, the energy used for plastic deformation decreased from 4.6 J to 3.15 J after 66 Wh/m^2^ of UV radiation. For PLA material samples, UV aging caused a decrease in the energy used for both elastic and plastic deformation, from 3.2 J to 2.57 J and from 4.21 J to 3.13 J, respectively.

The research results confirm the negative effect of UV exposure on the mechanical properties of PET material, as presented in [23], which studied polymer films exposed to UV radiation. Specifically, the properties of A-PET films decreased by about 15–25% when the UV irradiance reaches 98 Wh/m^2^.

## 4. Conclusions

This study conducted a comprehensive analysis of the mechanical properties of commonly used 3D printing materials to determine the effects of recycling and UV exposure on their mechanical properties and potential limitations for industrial use. Therefore, PLA, PET-G, and ABS materials produced from virgin materials were investigated, as well as recycled R_PET-G and R_ABS materials. This study investigated the essential mechanical properties of the printing materials, such as tensile strength, elongation at ultimate strength and fracture, and work performed in elastic and plastic tension regions during tensile testing. This study has also been extended to analyze the impact of UV exposure on new and recycled printing materials.

Specimens for mechanical property testing were printed from virgin (Standard) and recycled R-PLA, ABS, and PET-G materials. A comparison of new and recycled materials revealed that recycling affects mechanical properties, leading to changes in their values. Tensile test results showed that the relative elongation of recycled and 3D-printed materials decreased by 16–45%, indicating increased brittleness in the materials. The largest change in mechanical properties was observed when comparing new and recycled PET-G 3D-printed materials. A similar trend of reduced mechanical properties was observed in the impact test. The impact strength values of specimens made from recycled materials decreased, indicating that less energy is required to break the specimens. For materials PET-G and ABS, the reduction in this characteristic was more than 50%, while for PLA, it was only 6%.

The research results showed that the properties of recycled R-PLA, ABS, and PET-G materials change, making the materials more fragile. This should be considered when designing and printing products using recycled polymer materials.

UV aging tests were also performed on materials commonly used in 3D printers, such as PET-G, ABS, and PLA. The printed specimens were aged in UV aging chambers for up to 40 h, the irradiance of which was set at 1.65 W/m^2^. The obtained mechanical characteristics of the specimens were compared with the unaged specimens. It was found that the tensile stress of the all-aged materials was reduced, with PET-G and PLA tensile stress reduced by up to 17% and ABS by 5%. The plasticity properties of materials are well demonstrated by the impact of energy to break the specimen.

In particular, the UV aging affected the specimens of PET-G material, the total energy to completely break the specimen dropped as much as 61%, i.e., from 13.52 J to 5.26 J, where the total energy at breaking for ABS and PLA specimens decreased by 21% and 23%, respectively. The values of energy used for elastic deformation of PLA and PET-G specimens dropped slightly, while ABS did not change. However, the value of energy used for plastic deformation dropped very significantly only for PET-G material (about 85%), while for ABS it was 32%, and for PLA it was 26%.

In summary, all materials are sensitive to UV aging and mechanical properties deteriorate, but parts made of PET-G material should be especially protected from direct sunlight, because it degrades significantly enough, both tensile strength decreases, and it becomes brittle and cannot be used for parts if they are used under impact loads.

## Figures and Tables

**Table 1:** Three-dimensional printing equipment and properties.

Description	PLA	R_PLA	ABS	R_ABS	PET-G	R_PET-G
Initial layer nozzle temperature, °	215	215	255	255	235	235
Nozzle temperature, °	210	210	250	250	230	230
Printing bed temperature, initial layer, °	60	60	100	100	60	60
Printing bed temperature, °	60	60	110	110	60	60

**Table 2:** Values of tensile strength of 3D-printed polymer materials.

	PLA	R_PLA	ABS	R_ABS	PET-G	R_PET-G
Tensile strength (max), MPa	50.29	36.26	32.77	31.20	52.10	43.42
Standard deviation, MPa	1.75	0.91	0.51	2.04	0.05	0.98
Coefficient of variation (CV), %	3.48	2.55	1.49	6.54	0.09	2.26

**Table 3:** Values of relative elongation of 3D-printed polymer materials.

	PLA	R_PLA	ABS	R_ABS	PET-G	R_PET-G
Elongation at $\sigma^{max}$, %	3.04	2.51	2.84	2.36	7.01	4.48
Standard deviation, %	0.07	0.06	0.04	0.82	0.02	0.21
Coefficient of variation (CV), %	2.22	2.58	1.31	34.75	0.29	4.69

**Table 4:** The results of the energy required to break the specimens.

Material	Total Energy, J	Energy of Elastic Region, J	Energy of Plastic Region, J
PLA	4.62	2.89	1.73
R_PLA	3.59	2.07	1.51
ABS	5.55	2.06	3.50
R_ABS	5.27	2.55	2.73
PET-G	13.52	5.50	8.02
R_PET-G	4.80	4.61	0.19

**Table 5:** The impact test results.

	PLA	R_PLA	ABS	R_ABS	PET-G	R_PET-G
Charpy impact strength, kJ/m^2^	5.24	4.91	9.46	4.31	7.91	3.29
Standard deviation, kJ/m^2^	0.78	0.51	0.51	0.02	0.62	0.04
Coefficient of variation (CV), %	14.89	10.38	5.39	6.73	7.83	1.29

## Data Availability

The data presented in this study are available on request from the corresponding authors.
